# Supplementary material for: Callyspongiolide kills cells by inducing mitochondrial dysfunction via cellular iron depletion
Source: Commun Biol. 2021 Sep 23;4:1123. doi: 10.1038/s42003-021-02643-8 (PMC8460830; doi:10.1038/s42003-021-02643-8)
Supplement: Supplementary file 2 — Supplementary Information [file 42003_2021_2643_MOESM2_ESM.pdf]

< Supporting Information >

Callyspongiolide Kills Cells by Inducing Mitochondrial Dysfunction *via* Cellular Iron Depletion

Jaeyoung Ha[1] and Seung Bum Park[1,2,3]*

[1]*Department of Biophysics and Chemical Biology, Seoul National University, Seoul, 08826, Korea*

[2]*CRI Center for Chemical Proteomics, Department of Chemistry, Seoul National University, Seoul, 08826, Korea*

[3]*SPARK Biopharma, Inc., Seoul, 08791, Korea*

*correspondence: sbpark@snu.ac.kr

Table of contents

1	Supplementary Figures	------------------------------------	2
2	Supplementary Table	------------------------------------	31

1. Supplementary Figures

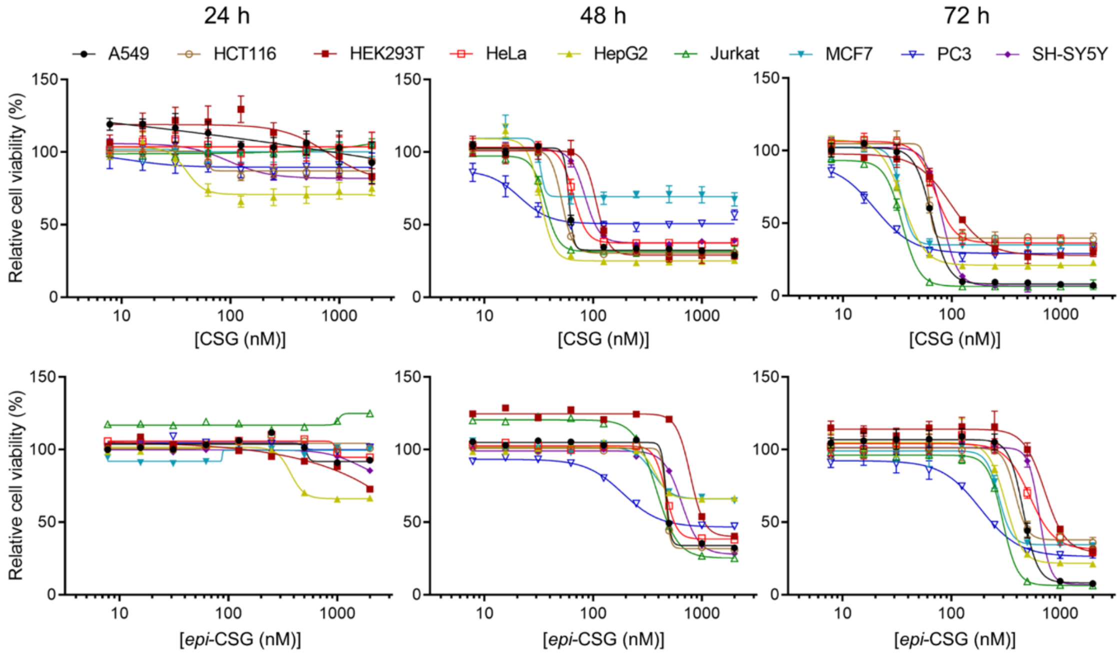

Supplementary Fig. 1 Dose-dependent cell viability upon treatment of (–)-CSG (upper) and 21-*epi*-(–)-CSG (lower) in human cancer cell lines (A549, HCT116, HEK293T, HeLa, Jurkat, HepG2, MCF7, PC3, and SH-SY5Y) for indicated times. Cell viability is presented as % relative to the DMSO-treated cells. Data represent the mean ± SD (n = 5–6).

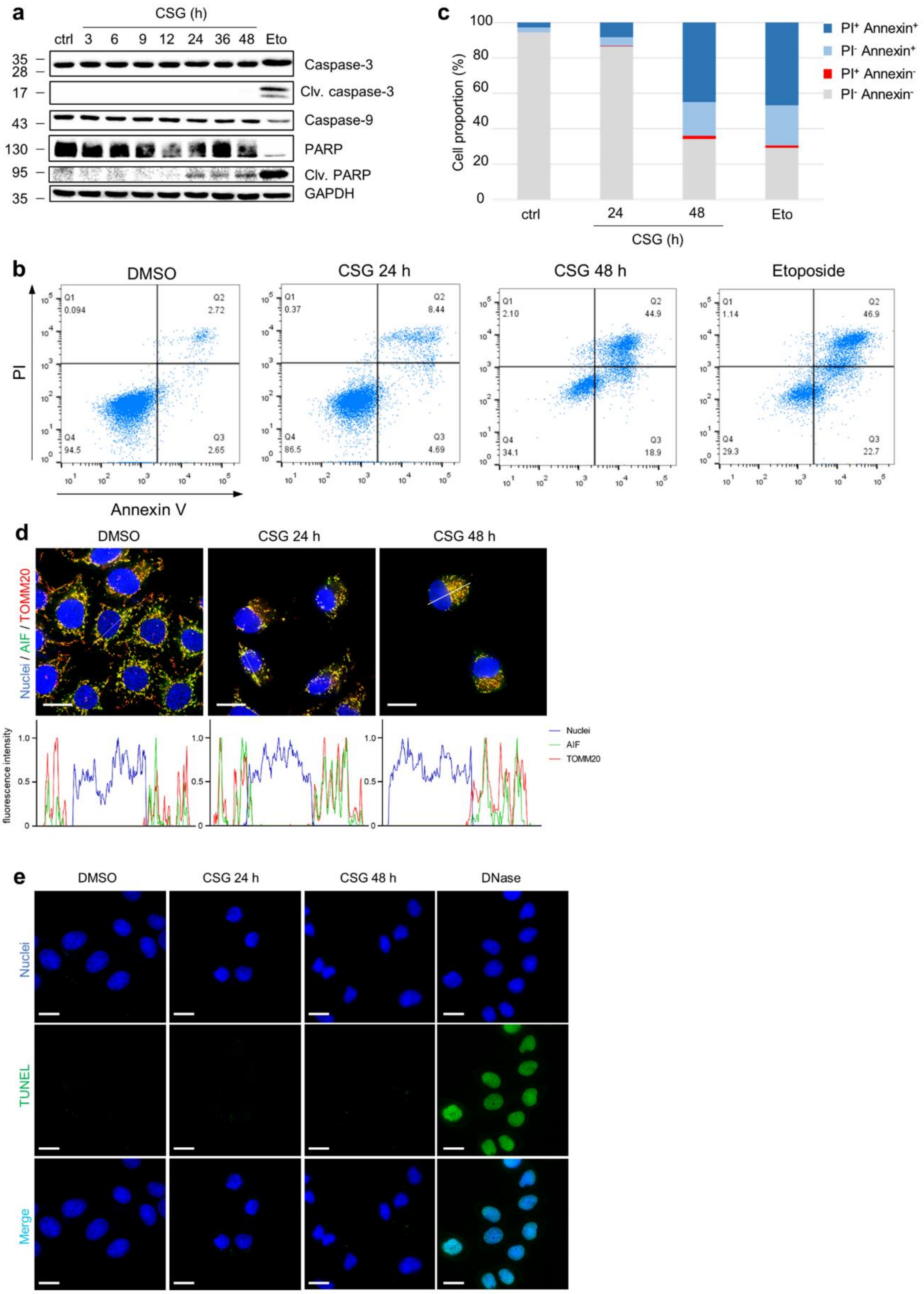

Supplementary Fig. 2. CSG kills cells via neither canonical apoptosis nor parthanatos.

(**a**) Immunoblotting of caspase-3, caspase-9, PARP in A549 cells upon treatment of CSG (200 nM) for indicated times or etoposide (Eto, 50 μM) for 48 h.

(**b, c**) FACS analysis of annexin V/PI staining in A549 cells upon treatment of CSG (200 nM) for indicated times or Eto (50 μM) for 48 h (**b**). Cell portion at each quadrant was quantified in (**c**).

(**d**) Representative immunofluorescence images of AIF (green) and TOMM20 (red) in A549 cells upon treatment of CSG (200 nM) for indicated times. Nuclei were stained with Hoechst 33342. Color profiling through white lines is presented below as % relative to the maximum. Scale bar, 10 μm.

(**e**) Representative images of TUNEL assay in A549 cells upon treatment of CSG (200 nM) for indicated times. DNase-treated condition was used as a positive control. Scale bar, 10 μm.

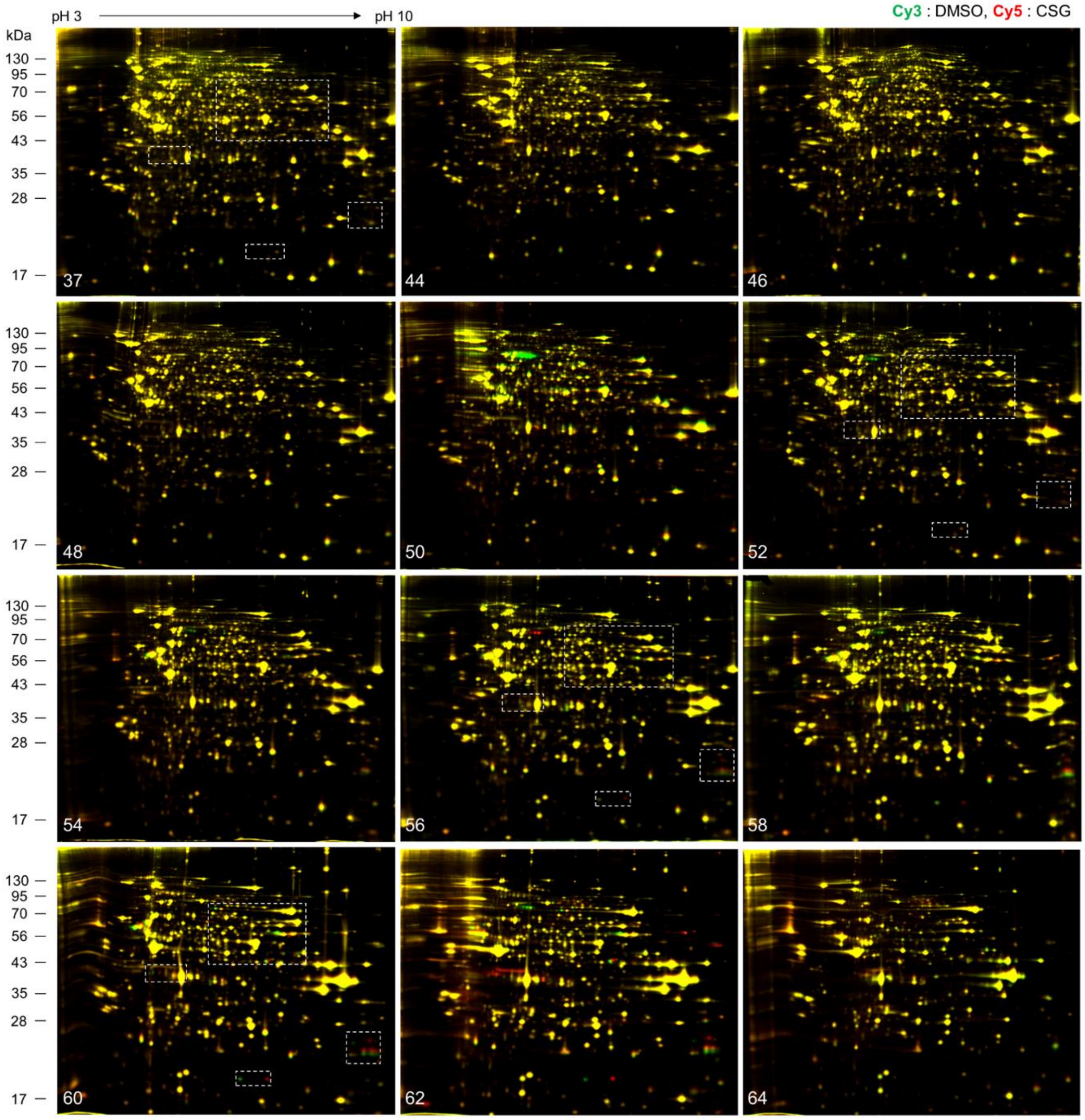

Supplementary Fig. 3 Representative images of TS-FITGE (pH 3–10) with CSG from 37 to 64 ºC at 2 ºC intervals in A549 cells. Overlaid images of the Cy3 channel (green, DMSO-treated proteome) and Cy5 channel (red, CSG-treated proteome). The magnified regions in **Fig. 2b–e** are indicated as the white-dotted squares.

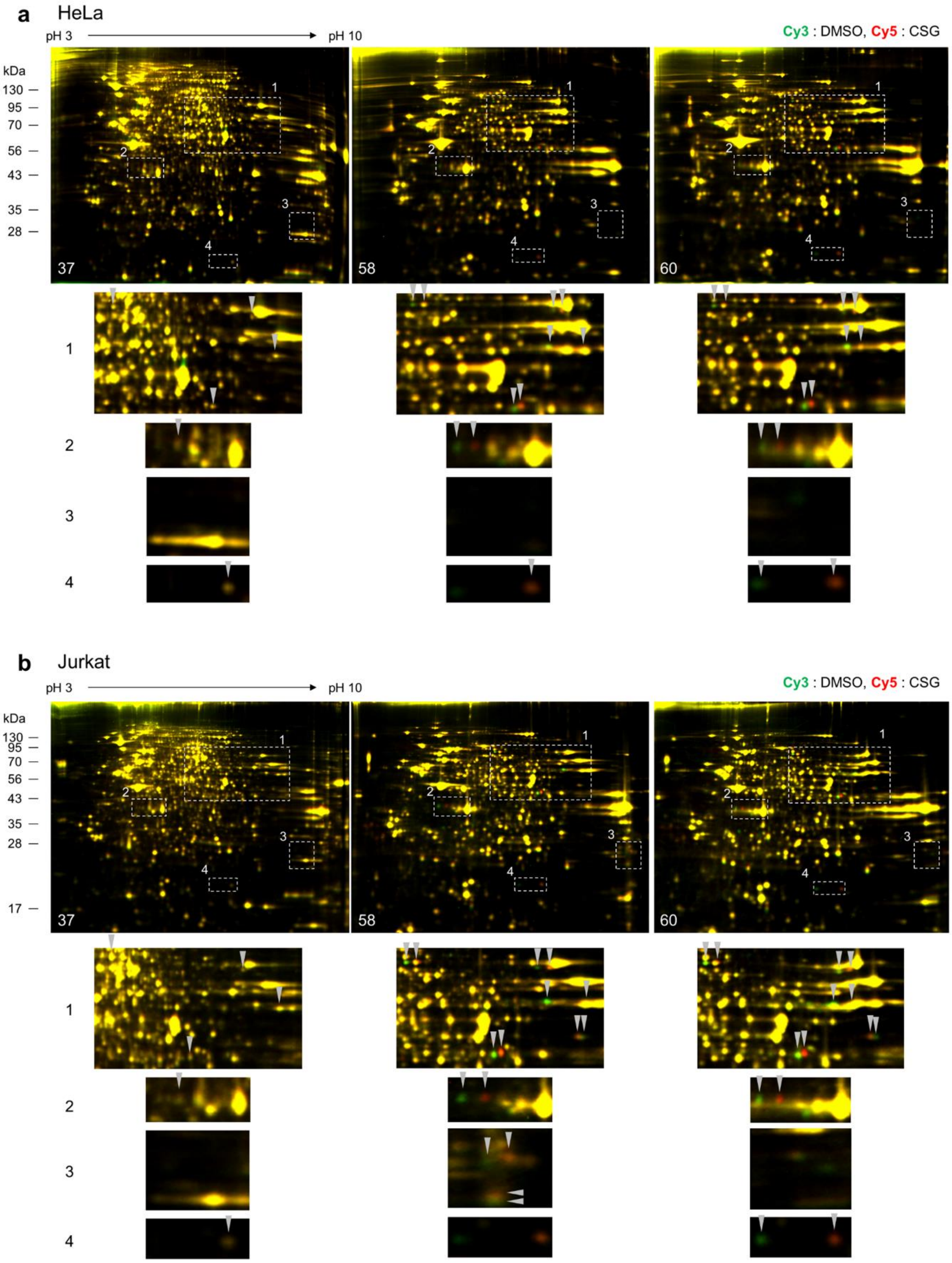

Supplementary Fig. 4 Representative images of TS-FITGE (pH 3–10) with CSG at 37, 58, and 60 ºC in HeLa human cervical cancer cells (**a**) or Jurkat human T lymphocyte cells (**b**). Overlaid images of the Cy3 channel (green, DMSO-treated proteome) and Cy5 channel (red, CSG-treated proteome). The same regions in **Fig. 2** and **Supplementary Fig. S3** are indicated as white-dotted squares 1–4.

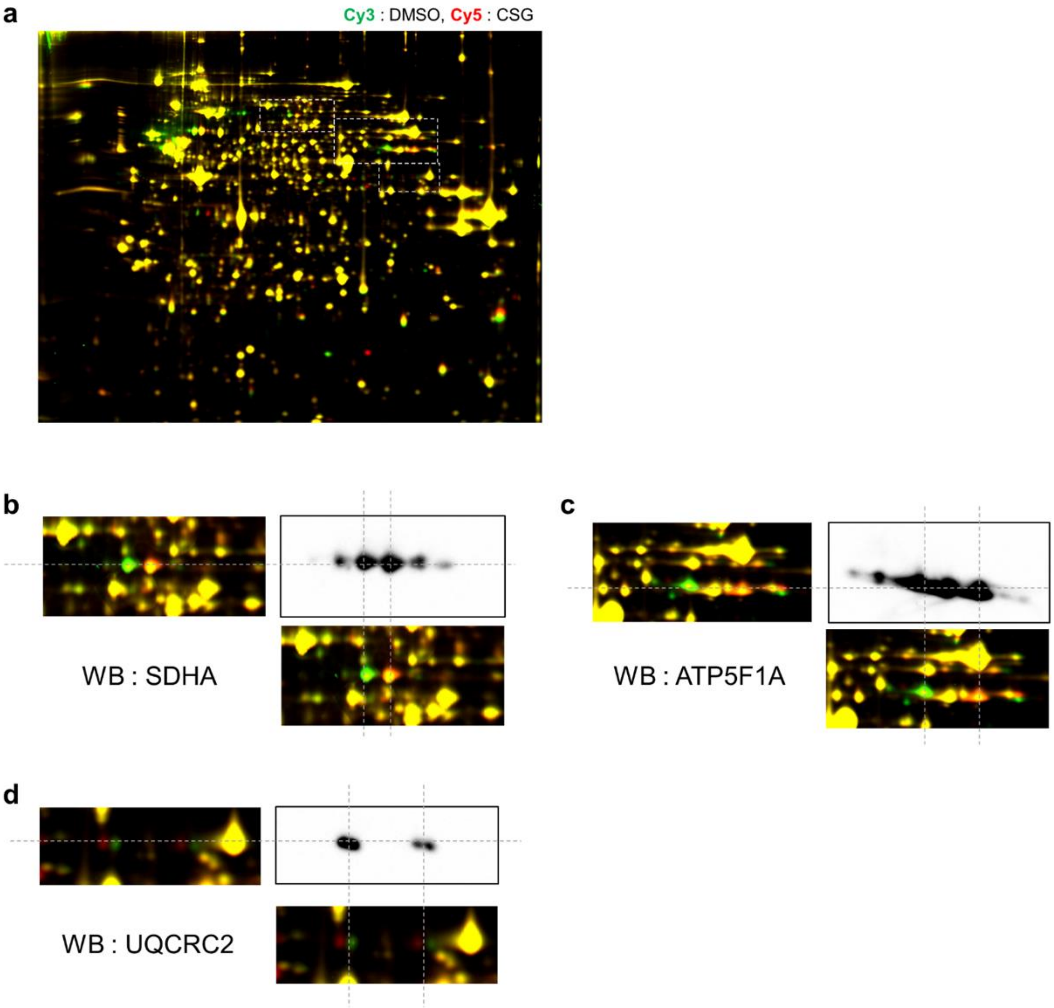

Supplementary Fig. 5 Spot validation of SDHA, ATP5F1A, and UQCRC2 on 2-D gels.

(**a**) Whole 2-D gel image used for immunoblotting of specific region containing each protein spot. The regions noted as white-dotted squares were excised and subjected to immunoblot of SDHA, ATP5F1A, and UQCRC2, respectively. Overlaid images of the Cy3 channel (green, DMSO-treated proteome) and Cy5 channel (red, CSG-treated proteome).

(**b–d**) Immunoblotting results of SDHA (**b**), ATP5F1A (**c**), and UQCRC2 (**d**) on 2-D gels.

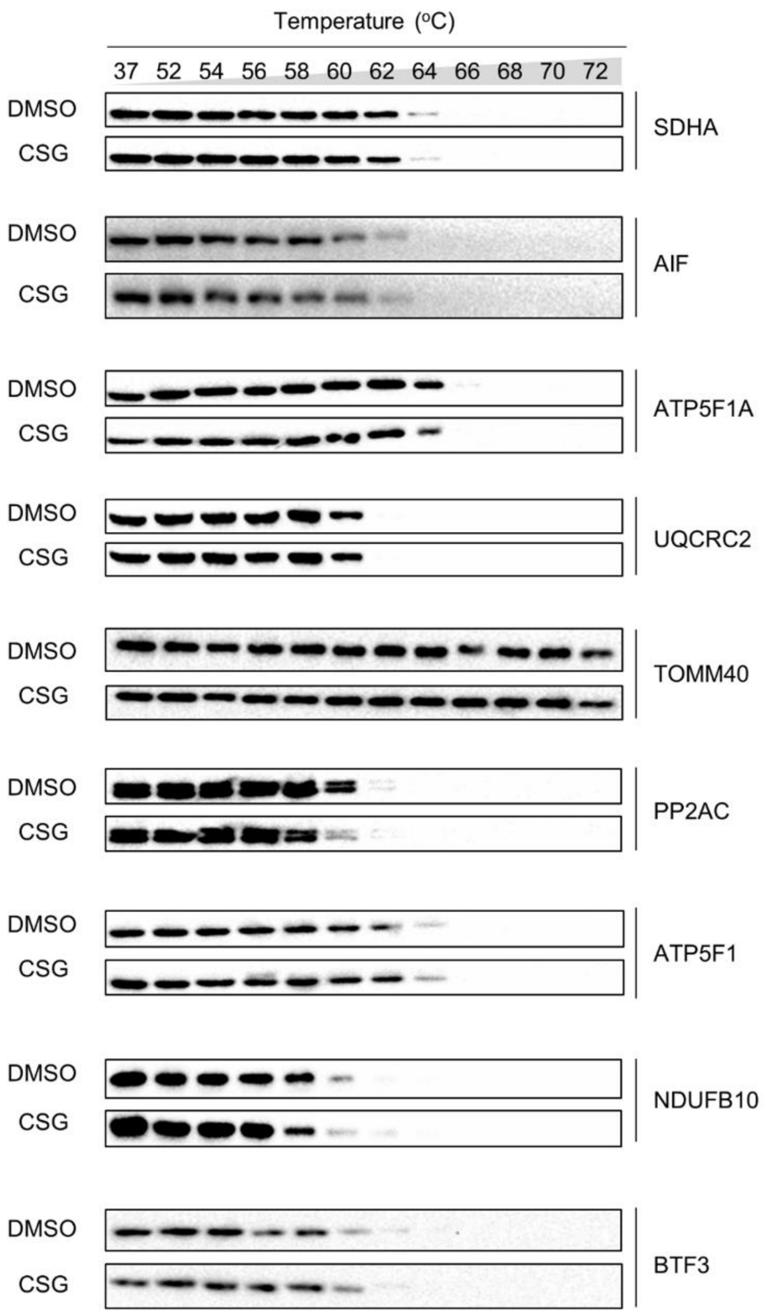

Supplementary Fig. 6 Representative cellular thermal stability shift assay (CETSA) results of CSG-treated A549 cells toward the proteins obtained from TS-FITGE experiments.

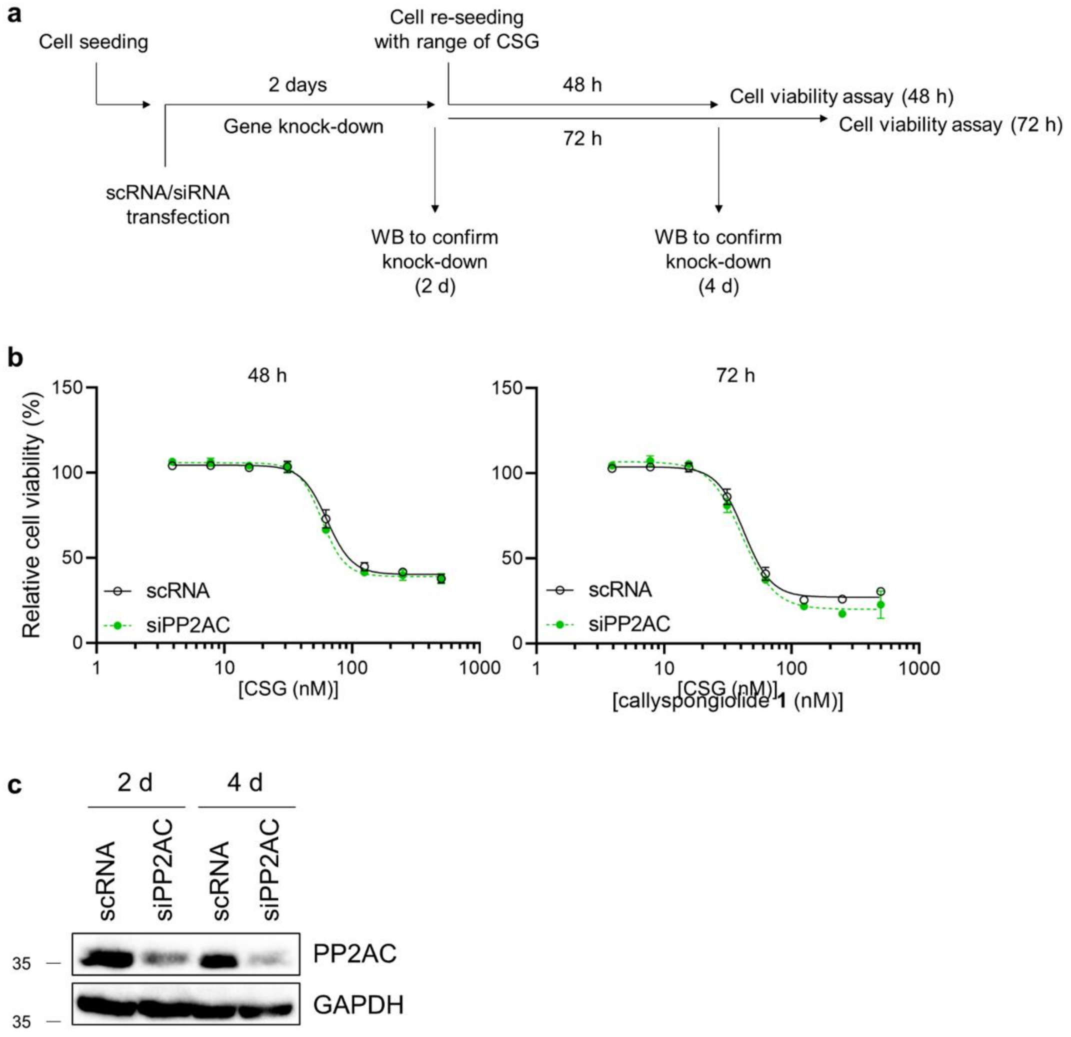

Supplementary Fig. 7 CSG-mediated cell-death potency was not altered upon PP2AC knockdown in the cells.

(**a**) Simplified scheme for this experiment.

(**b**) Dose-dependent cell viability upon treatment of CSG for indicated times in A549 cells upon PP2AC knockdown. Cell viability is presented as % relative to the DMSO-treated cells. Data represent the mean ± SD (n = 3).

(**c**) Immunoblotting of PP2AC to confirm the knockdown of target gene.

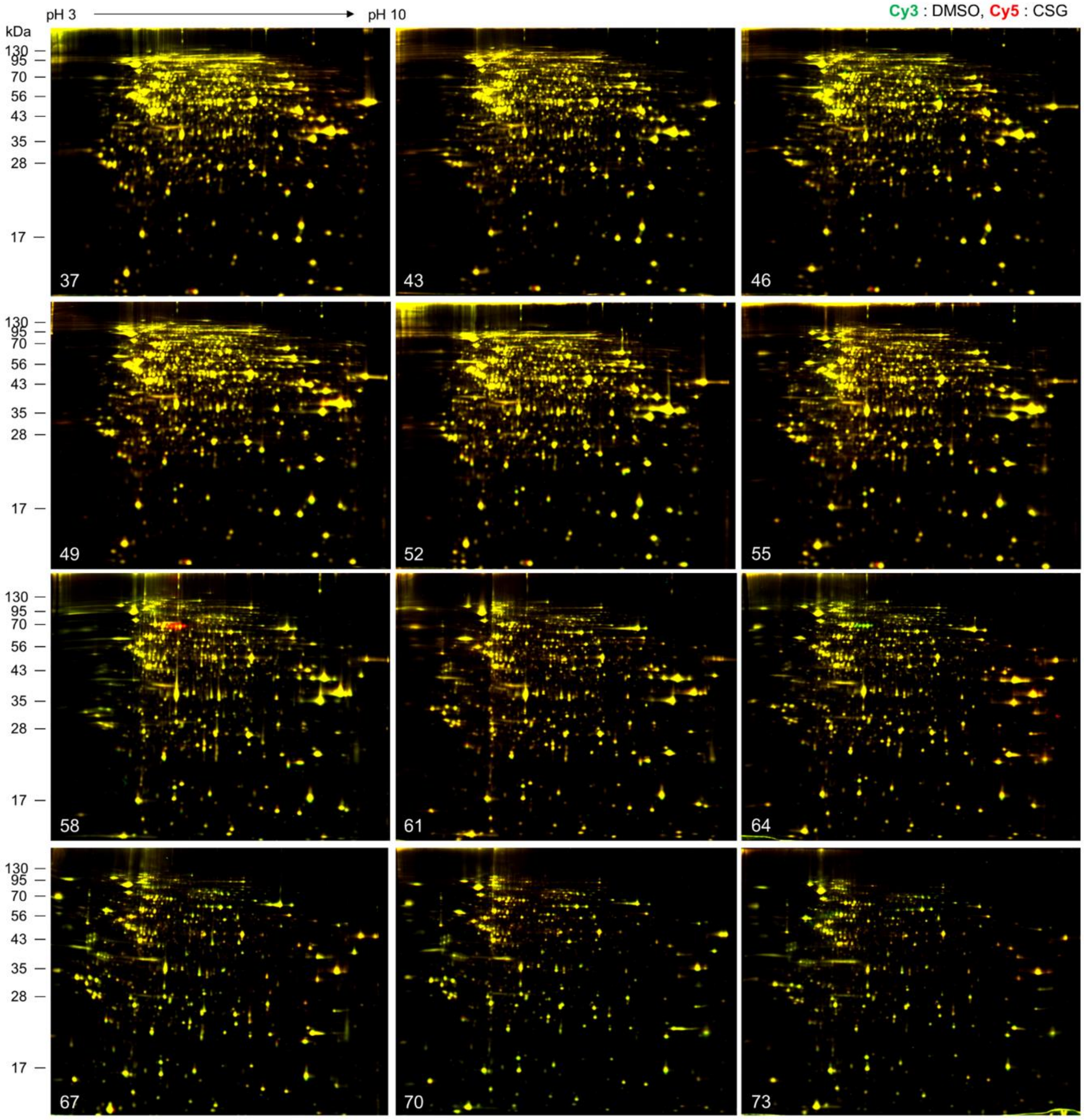

Supplementary Fig. 8 Representative images of TS-FITGE (pH 3–10) with CSG from 37 to 73 ºC at 3 ºC intervals in A549 cell lysate. Overlaid images of the Cy3 channel (green, DMSO-treated proteome) and Cy5 channel (red, CSG-treated proteome).

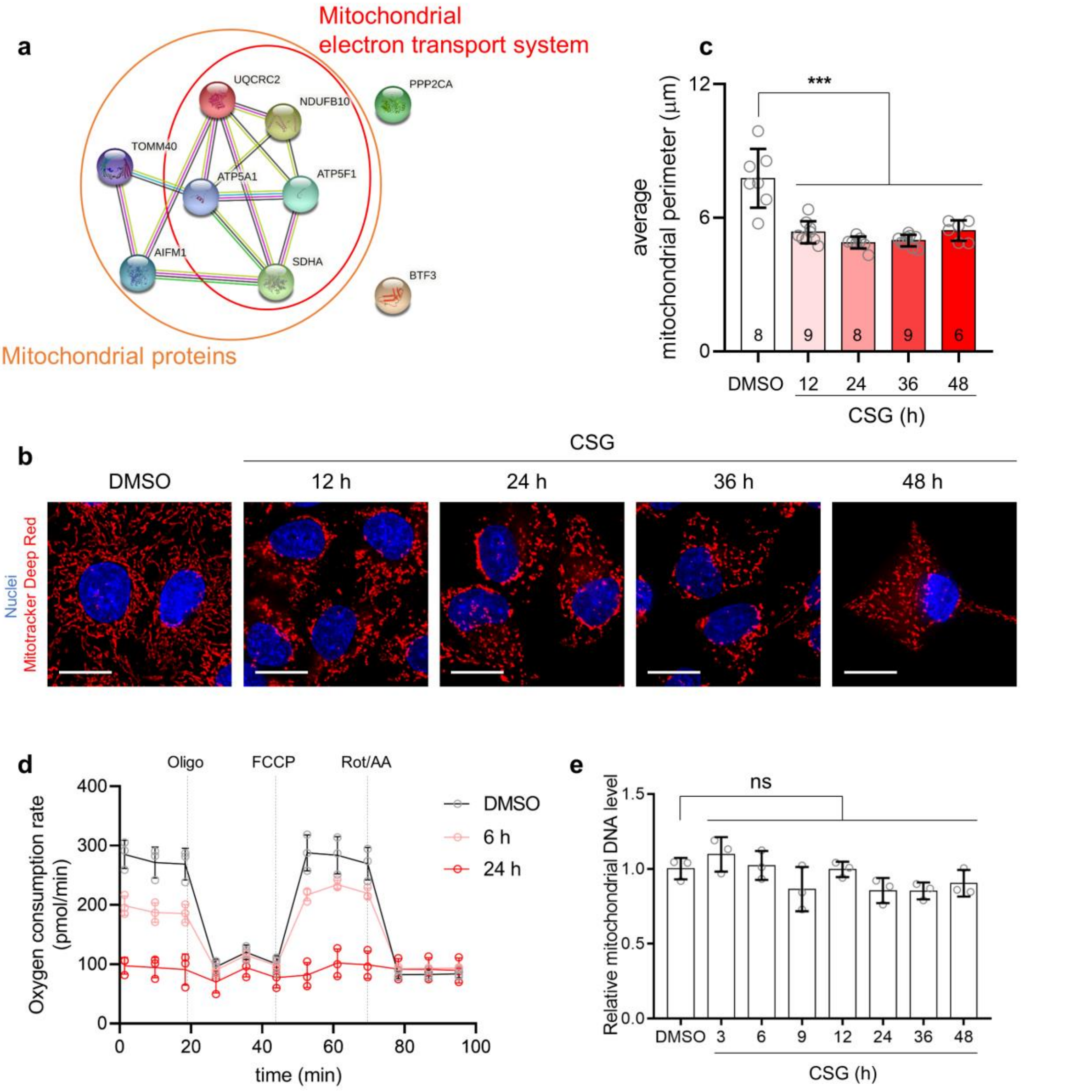

Supplementary Fig. 9 CSG has an impact on mitochondrial morphology and respiratory function.

(**a**) Protein network analysis of the proteins obtained from TS-FITGE experiments using STRING.

(**b**) Representative live-cell fluorescence imaging of mitochondria in A549 cells using Mitotracker Deep Red following treatment with CSG (200 nM) for indicated times. Nuclei were stained with Hoechst 33342. Scale bar, 10 μm.

(**c**) Quantification of the mitochondrial perimeter in (**b**). Data represent the mean ± SD (the number of quantified cells is indicated under each bar). ****P* < 0.001, one-way ANOVA with Bonferroni's *post hoc* test.

(**d**) Real-time bioenergetic analysis using Seahorse XF analyzer in A549 cells upon treatment of CSG (200 nM) for indicated times. Mitochondrial respiration was measured as oxygen consumption rate (pmol/min). Data represent the mean ± SD (n = 3).

(**e**) Relative mitochondrial DNA levels in A549 cells upon treatment of CSG (200 nM) for indicated times. Mitochondrial DNA levels were normalized to β-actin DNA levels. Data represent the mean ± SD (n = 3). ns: *P* > 0.05, one-way ANOVA with Dunnett's *post hoc* test.

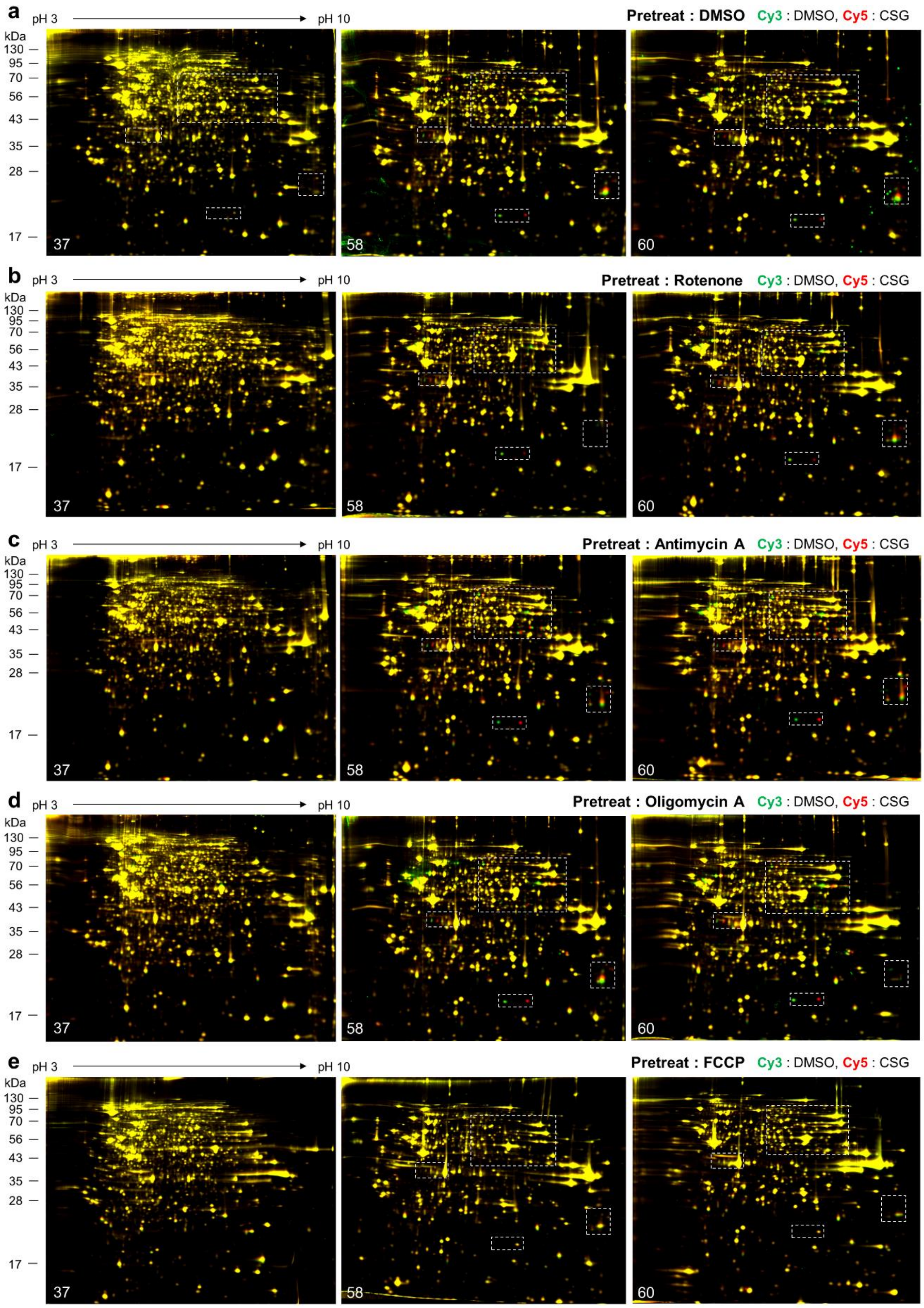

Supplementary Fig. 10 Representative images of TS-FITGE (pH 3–10) with CSG upon pre-treatment of DMSO (**a**), rotenone (**b**), antimycin A (**c**), oligomycin A (**d**), or FCCP (**e**) at 37, 58, and 60 ºC in A549 cells. Overlaid images of the Cy3 channel (green, DMSO-treated proteome) and Cy5 channel (red, CSG-treated proteome). The magnified regions 1–4 in **Fig. 3a–c** and the same regions in **Fig. 2** and **Supplementary Fig. 3** are indicated as white-dotted squares.

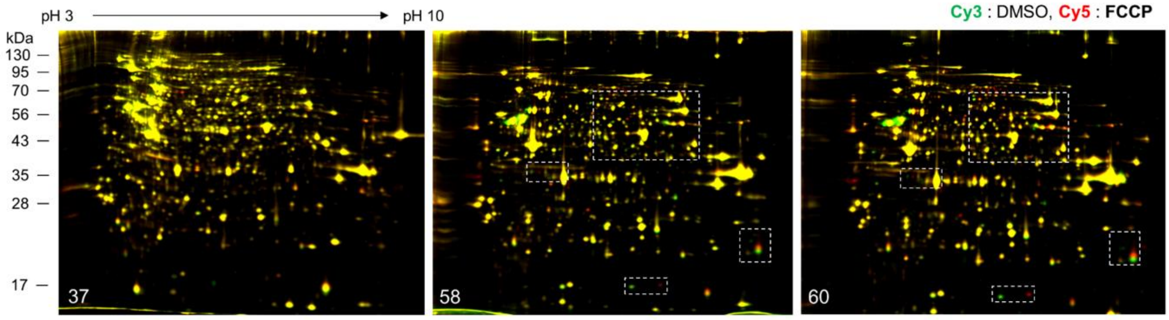

Supplementary Fig. 11 Representative images of TS-FITGE (pH 3–10) with FCCP at 37, 58, and 60 ºC in A549 cells. Overlaid images of the Cy3 channel (green, DMSO-treated proteome) and Cy5 channel (red, FCCP-treated proteome). The magnified regions 1–4 in **Fig. 3d** are indicated as the white-dotted squares.

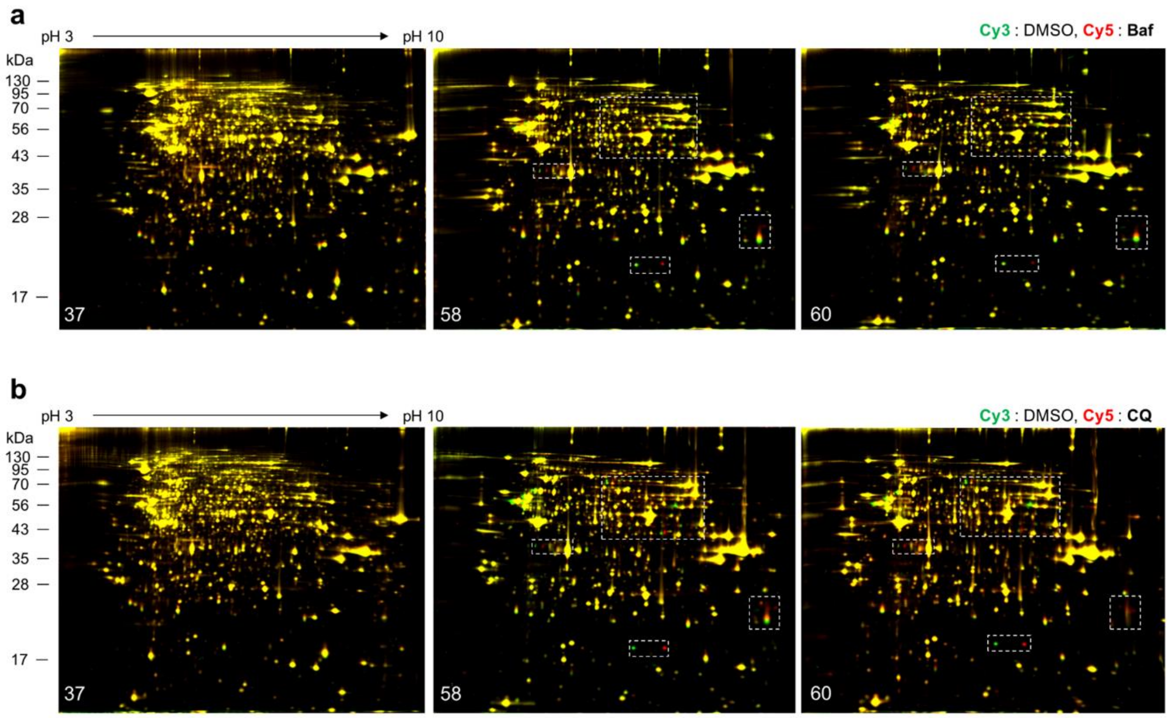

Supplementary Fig. 12 Representative images of TS-FITGE (pH 3–10) with bafilomycin A1 (Baf) (**a**) or chloroquine (CQ) (**b**) at 37, 58, and 60 ºC in A549 cells. Overlaid images of the Cy3 channel (green, DMSO-treated proteome) and Cy5 channel (red, Baf- or CQ-treated proteome, respectively). The same regions in **Fig. 2** and **Supplementary Fig. 3** are indicated as white-dotted squares.

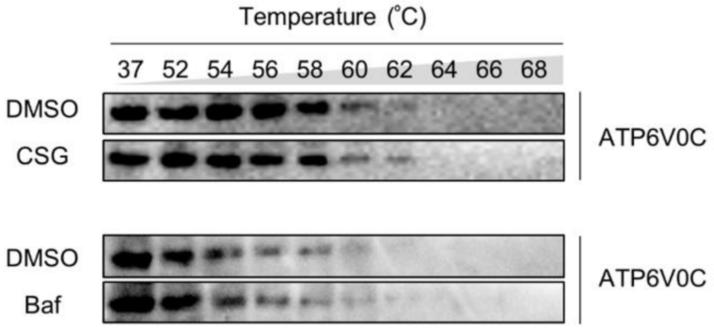

Supplementary Fig. 13 Representative CETSA results of CSG- or Baf-treated A549 cells toward ATP6V0C.

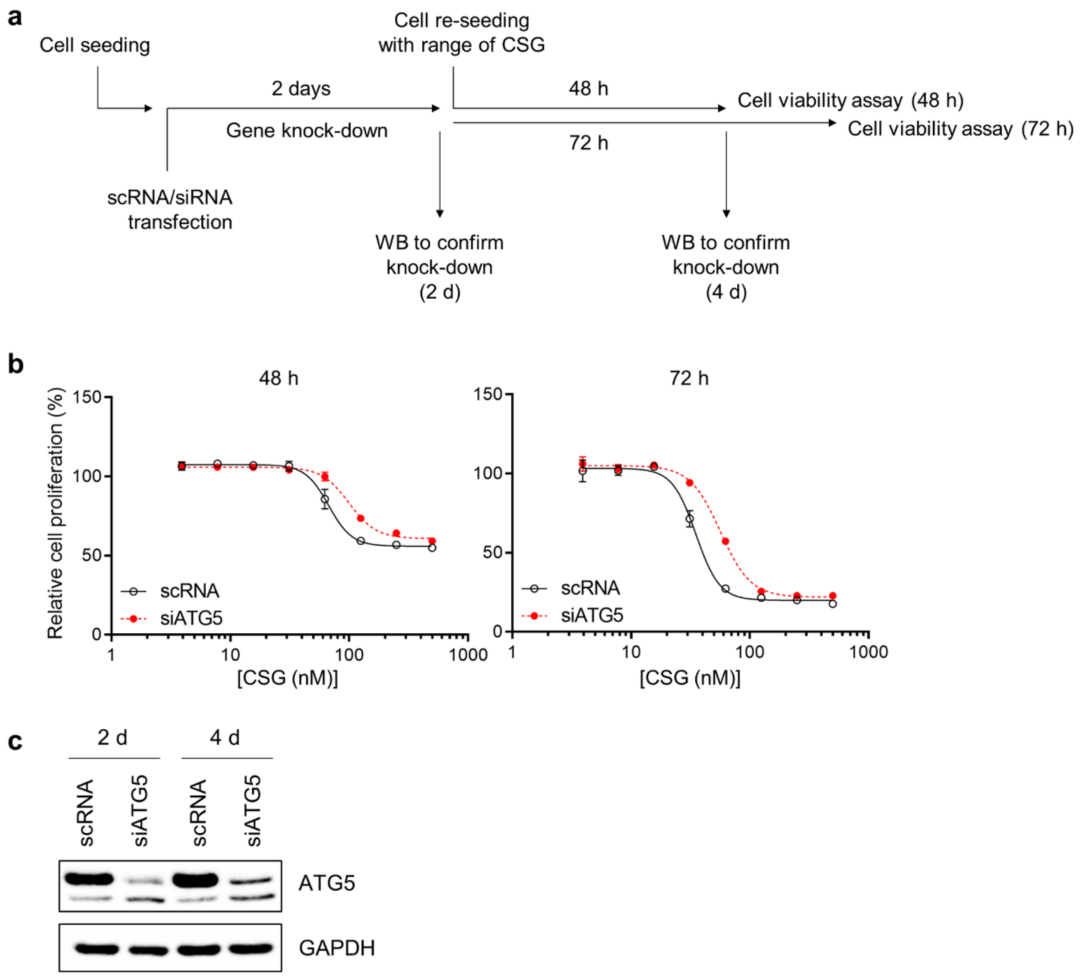

Supplementary Fig. 14 CSG-mediated cell death was not significantly affected upon ATG5 knockdown.

(**a**) Simplified scheme for this experiment. The detailed method is available in **Supplementary Methods**.

(**b**) Dose-dependent cell viability following treatment with CSG for indicated times in A549 cells upon ATG5 knockdown. Cell viability is presented as % relative to the DMSO-treated cells. Data represent the mean ± SD (n = 3).

(**c**) Immunoblotting of ATG5 to confirm the knockdown of target gene.

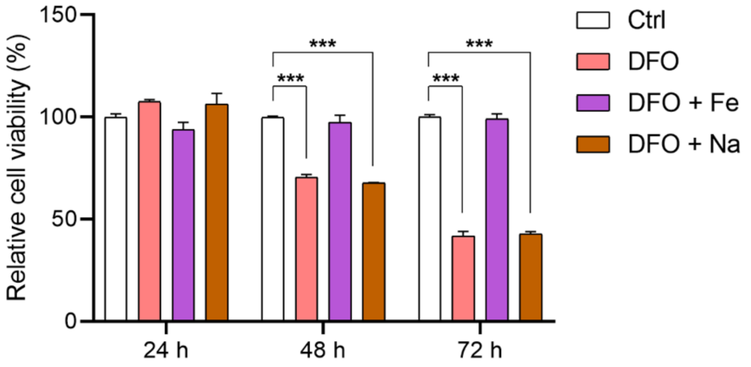

Supplementary Fig. 15 Cytotoxicity induced by the iron chelator deferoxamine (DFO) and its viability rescue upon iron citrate supplementation, but not by sodium citrate. DFO (100 μM) was treated to A549 cells in the absence or presence of ferric citrate (200 μM) or sodium citrate (200 μM) for indicated times. Cell viability is presented as % relative to the vehicle-treated cells. Data represent the mean ± SD (n = 3). ***$P < 0.001$. one-way ANOVA with Dunnett's *post hoc* test.

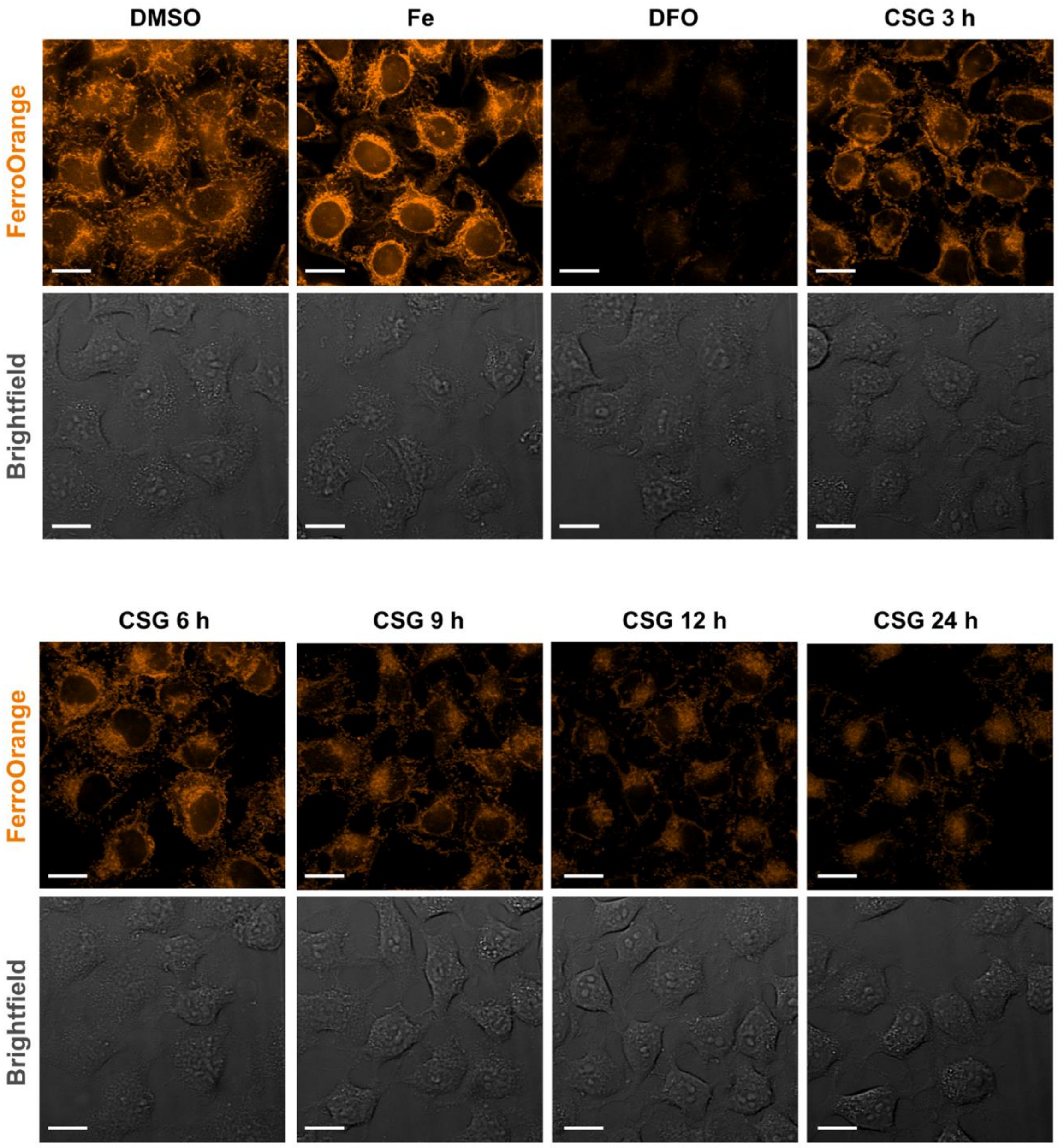

Supplementary Fig. 16 Representative live-cell fluorescent images in A549 cells with FerroOrange staining and brightfield images upon treatment of ferric citrate (Fe, 200 μM), deferoxamine (DFO, 100 μM) for 24 h, or CSG (200 nM) for indicated times. Scale bar, 10 μm.

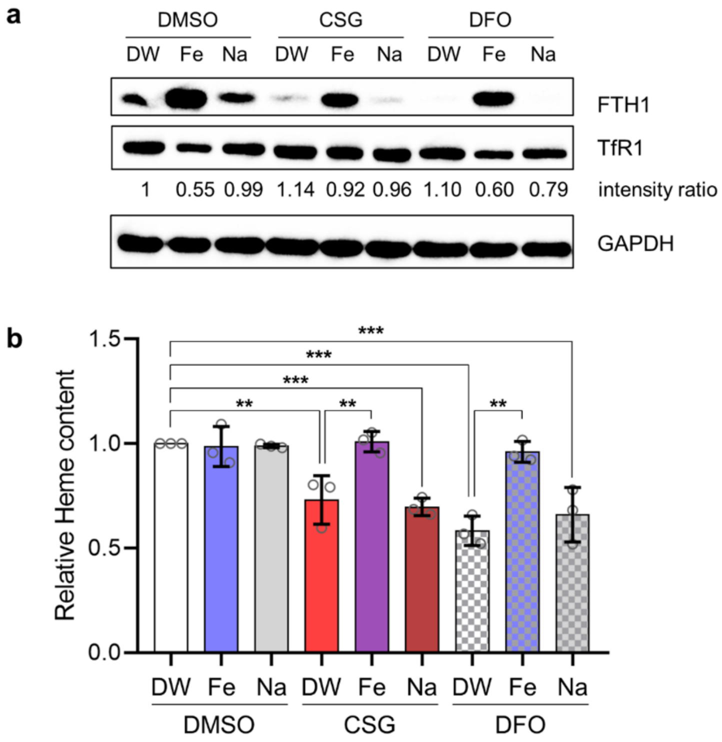

Supplementary Fig. 17 Cellular responses to cellular iron deficiency induced by CSG treatment.

(**a**) Immunoblotting of FTH1, TfR1 in A549 cells upon treatment with CSG (200 nM) or DFO (100 µM), and supplementation with or without iron citrate (200 µM) or sodium citrate (200 µM) for 24 h.

(**b**) Heme content assay with fluorometric method in A549 cells upon treatment with CSG (200 nM) or DFO (100 µM), and supplementation with or without iron citrate (200 µM) or sodium citrate (200 µM) for 24 h. Relative heme content is presented as ratio to the DMSO- and DW-treated cells. Data represent the mean ± SD (n = 3). ** $P < 0.01$, ***$P < 0.001$. one-way ANOVA with Dunnett's *post hoc* test.

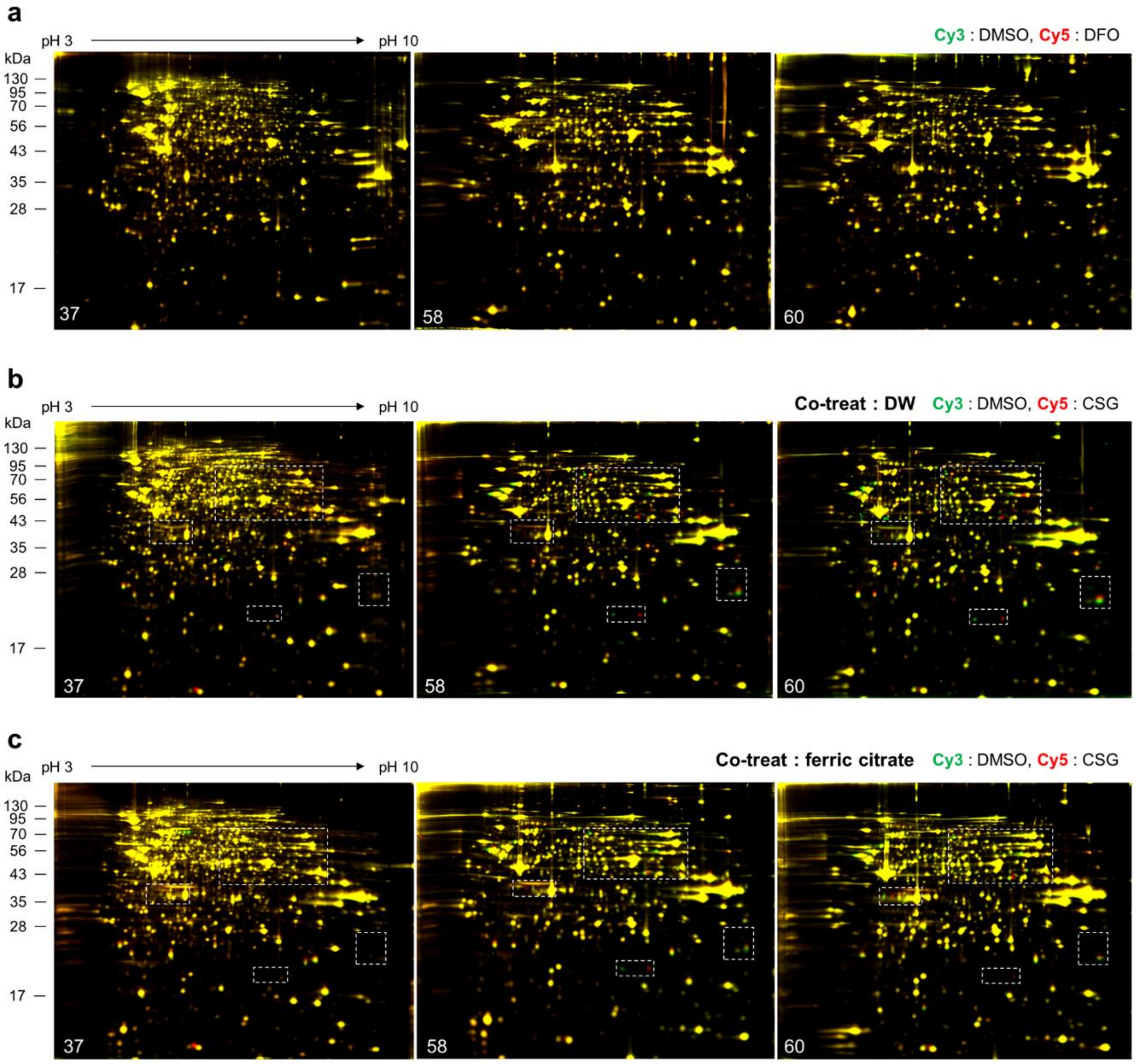

Supplementary Fig. 18 Representative images of TS-FITGE

(**a**) Representative images of TS-FITGE (pH 3–10) with deferoxamine (DFO) at 37, 58, and 60 ºC in A549 cells. Overlaid images of the Cy3 channel (green, DMSO-treated proteome) and Cy5 channel (red, DFO-treated proteome).

(**b, c**) Representative images of TS-FITGE (pH 3–10) with CSG upon co-treatment with distilled water (DW, **b**) or ferric citrate (Fe, **c**) at 37, 58, and 60 ºC in A549 cells. Overlaid images of the Cy3 channel (green, DMSO-treated proteome) and Cy5 channel (red, CSG-treated proteome). The same regions as in **Fig. 2** and **Supplementary Fig. 3** are indicated as white-dotted squares.

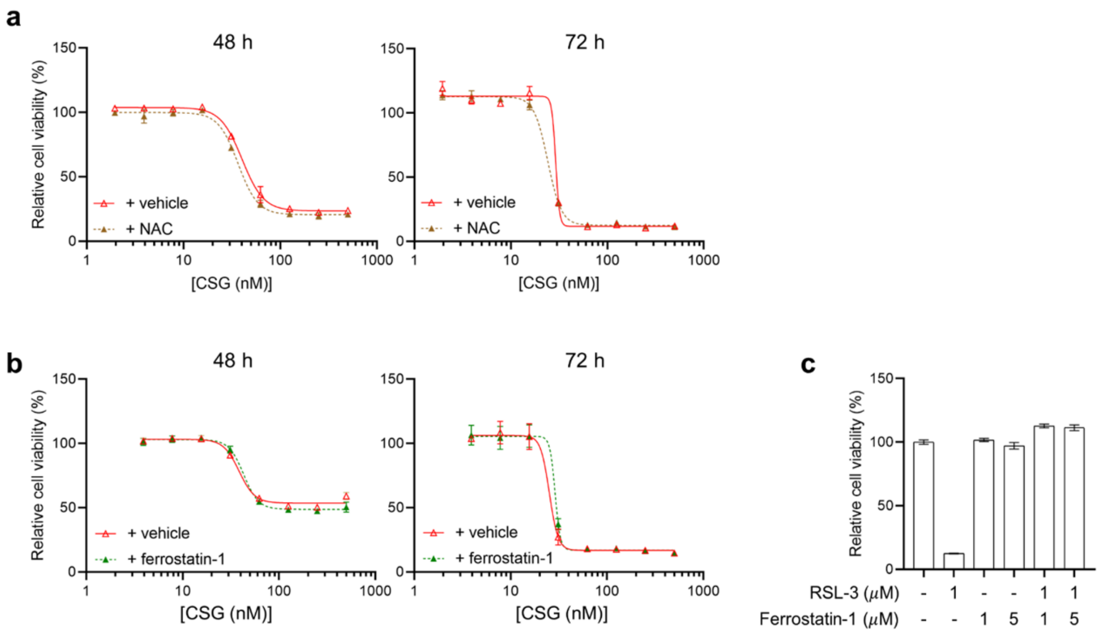

Supplementary Fig. 19 CSG-mediated cell death was not mediated by elevated ROS or ferroptosis.

(**a**) Dose-dependent cell viability of A549 cells upon treatment with CSG in the absence or presence of *N*-acetylcysteine (NAC, 1 mM) for indicated times. Cell viability is presented as % relative to the vehicle-treated cells. Data represent the mean ± SD (n = 3).

(**b**) Dose-dependent cell viability of A549 cells following treatment with CSG in the absence or presence of ferrostatin-1 (1 μM) for indicated times. Cell viability is presented as % relative to the vehicle-treated cells. Data represent the mean ± SD (n = 3).

(**c**) Cell viability following treatment of A549 cells with RSL3 in the absence or presence of ferrostatin-1 for 24 h. Cell viability is presented as % relative to the vehicle-treated cells. Data represent the mean ± SD (n = 6).

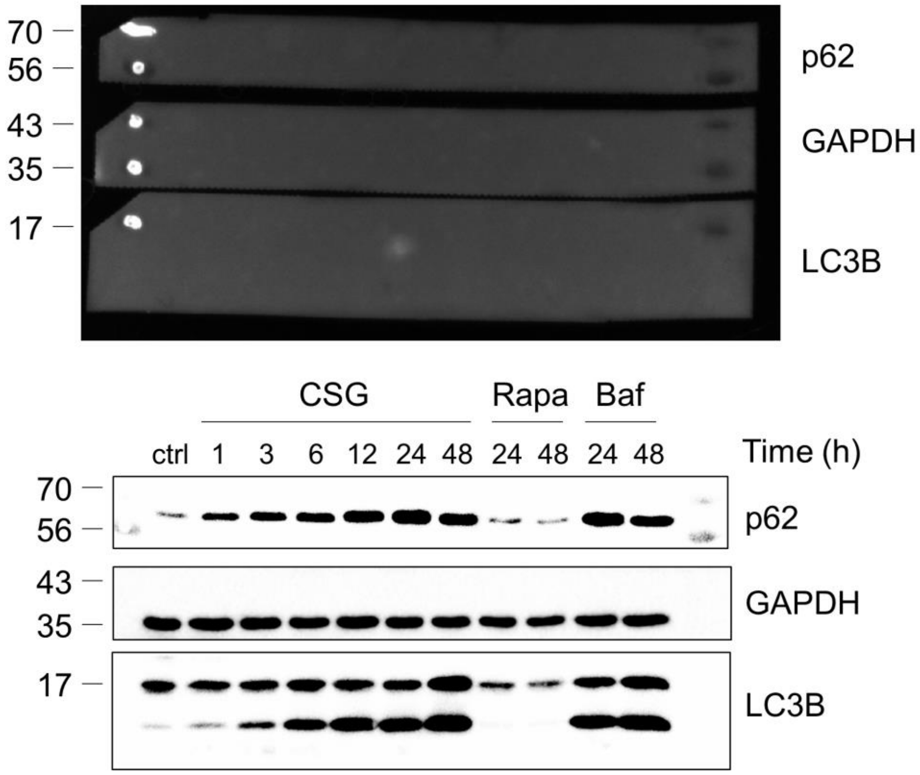

Supplementary Fig. 20 Uncropped immunoblots of **Fig. 4d**.

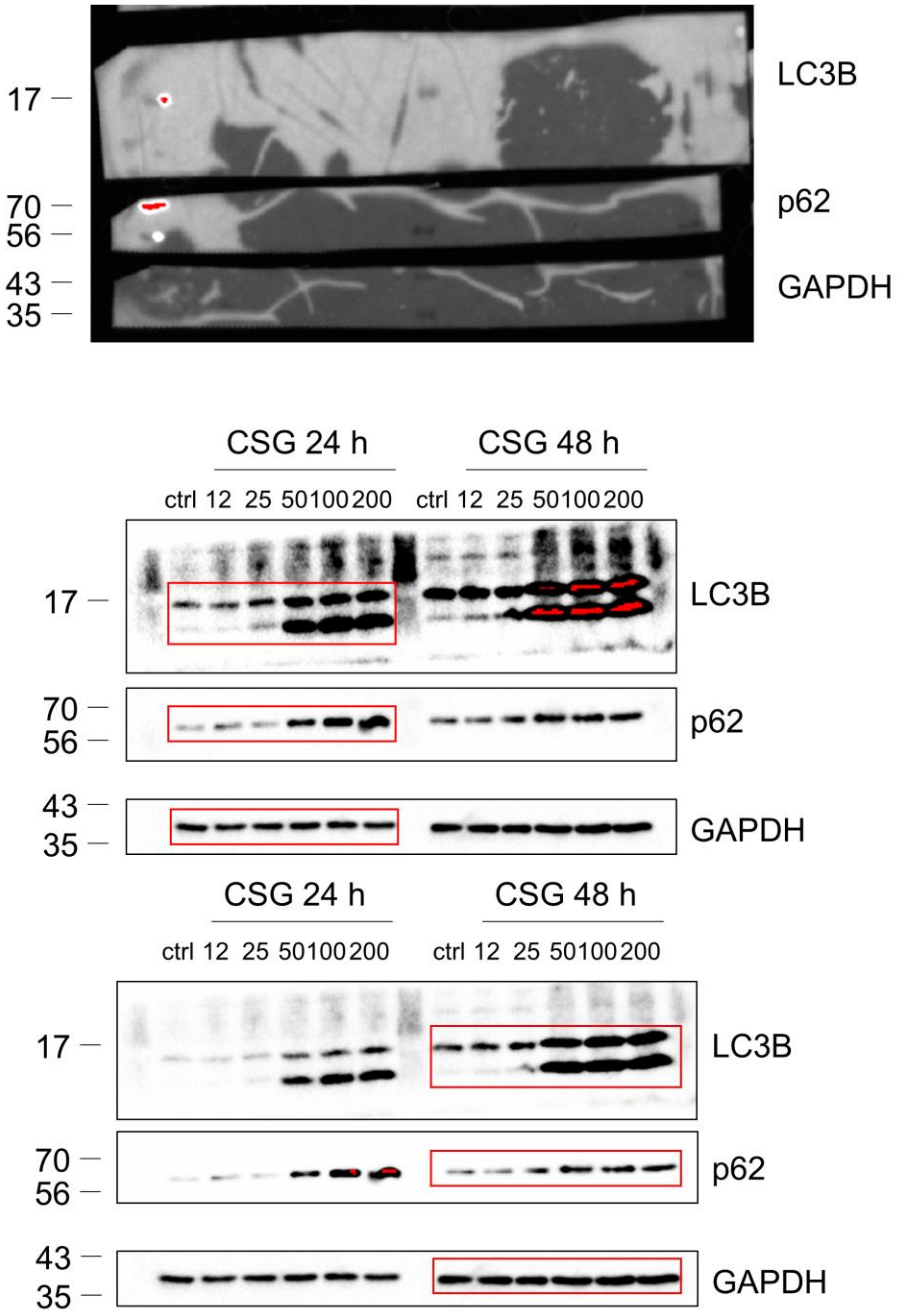

Supplementary Fig. 21 Uncropped immunoblots of **Fig. 4e**. Red rectangle means the cropped regions.

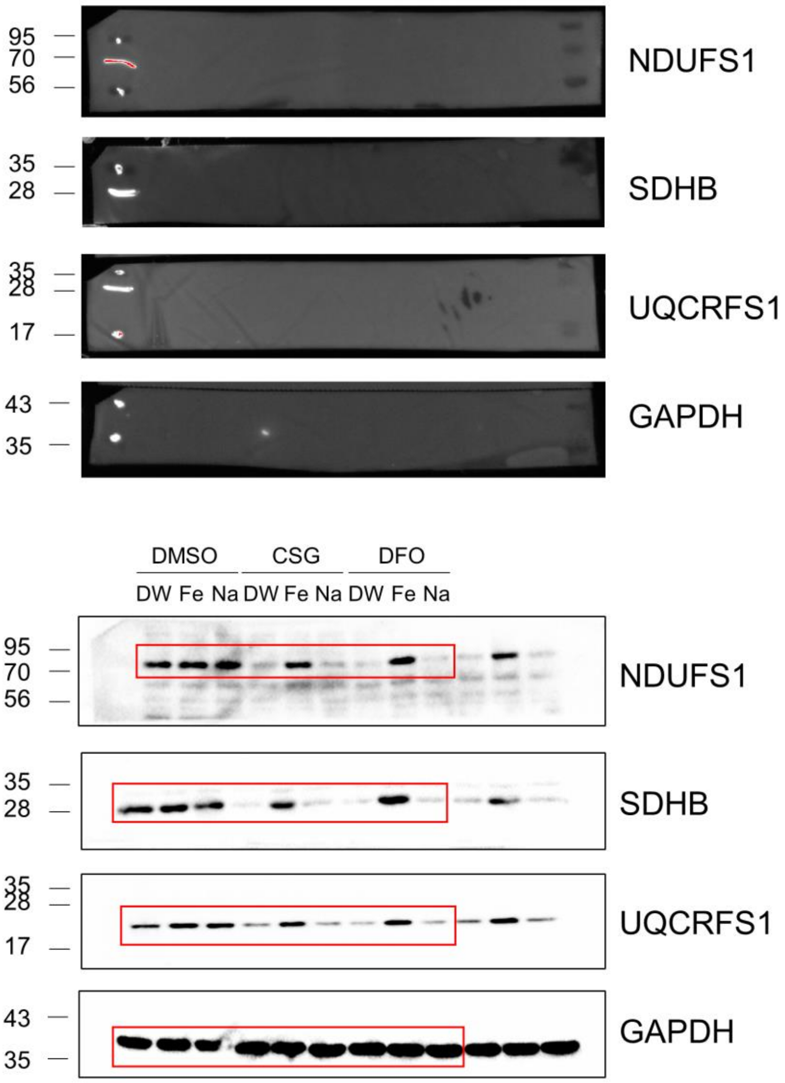

Supplementary Fig. 22 Uncropped immunoblots of **Fig. 5e**. Red rectangle means the cropped regions.

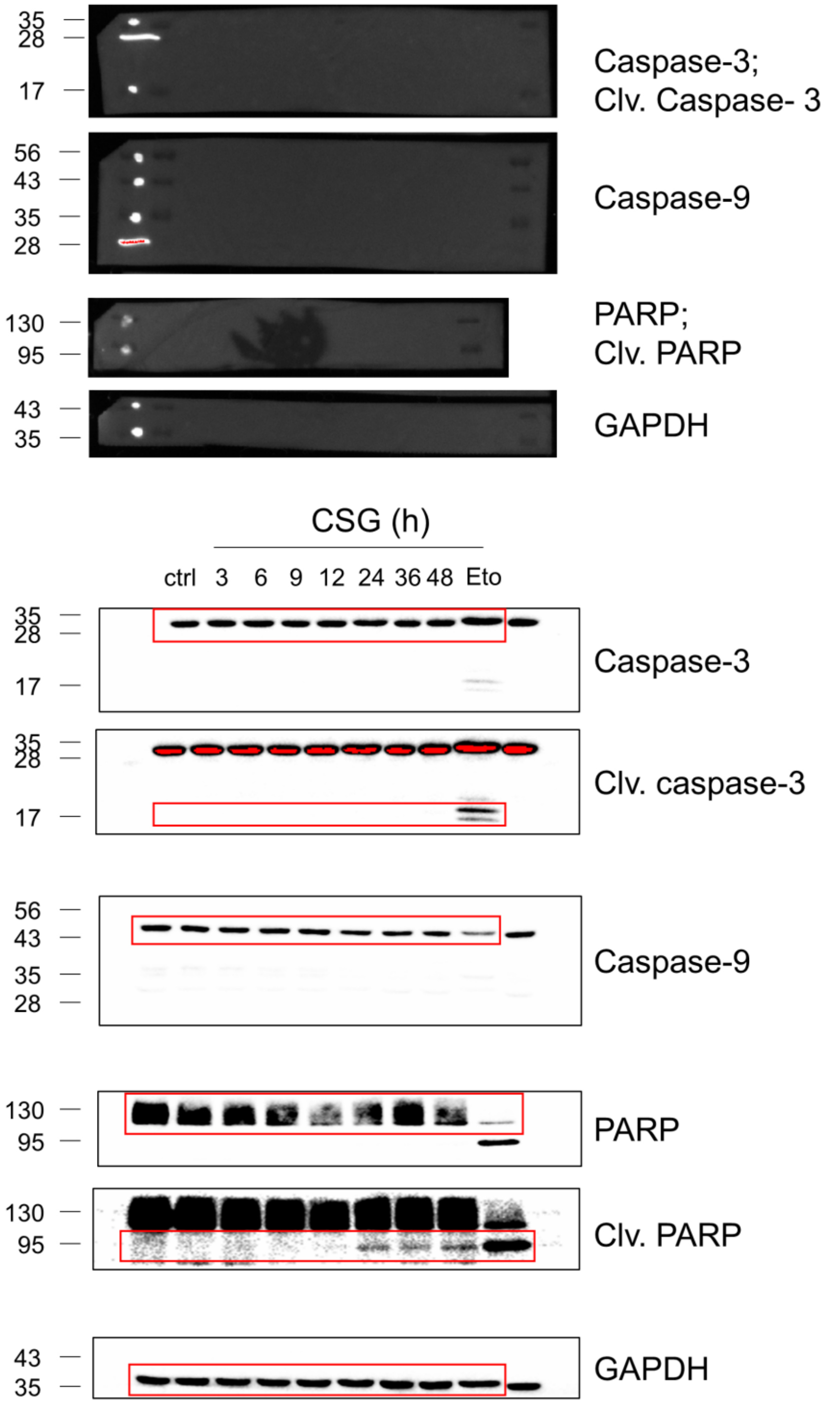

Supplementary Fig. 23 Uncropped immunoblots of **Supplementary Fig. 2a**. Red rectangle means the cropped regions.

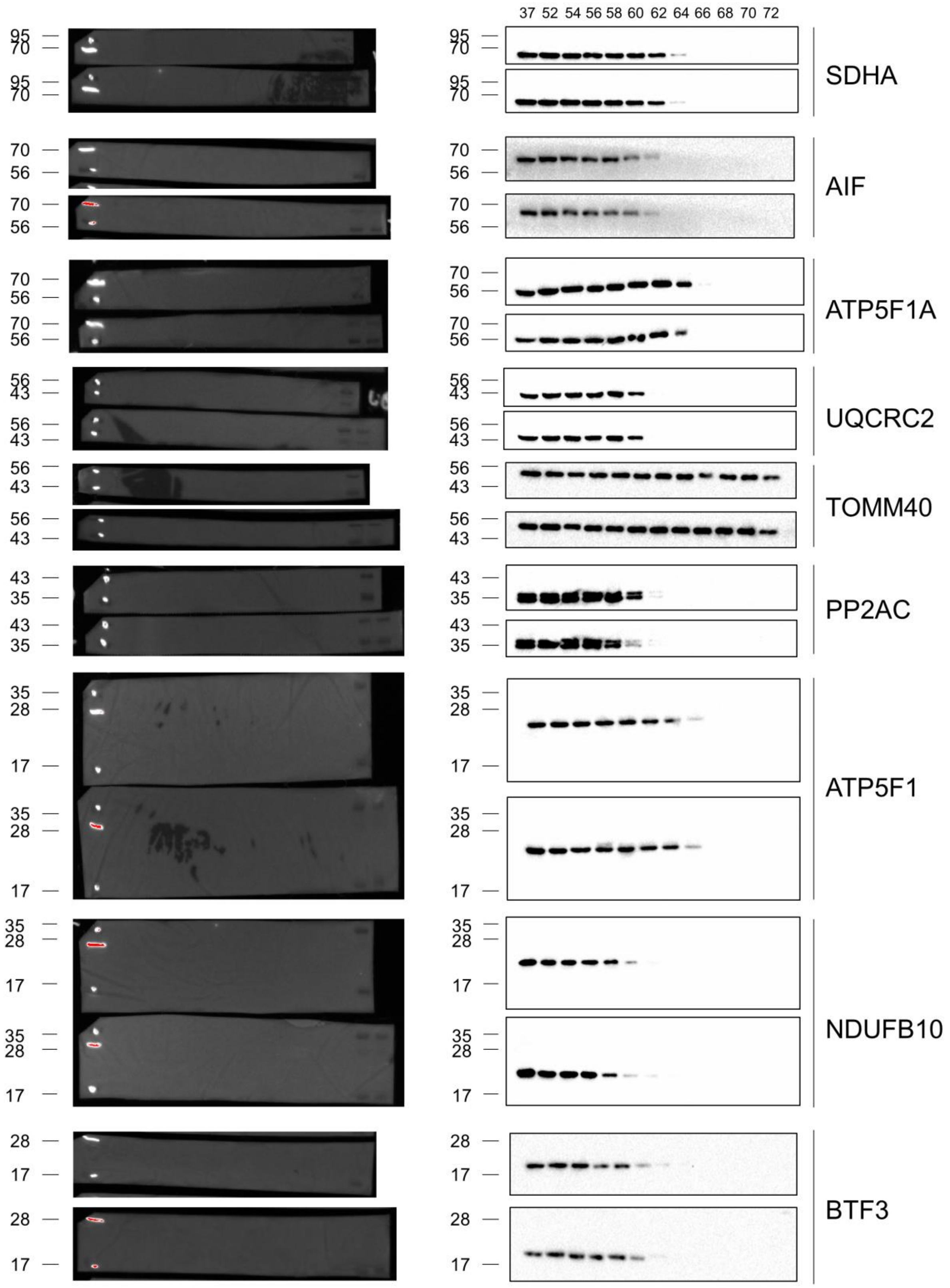

Supplementary Fig. 24 Uncropped immunoblots of **Supplementary Fig. 6**. Upper blots mean DMSO-treated condition, and lower blot mean CSG-treated condition.

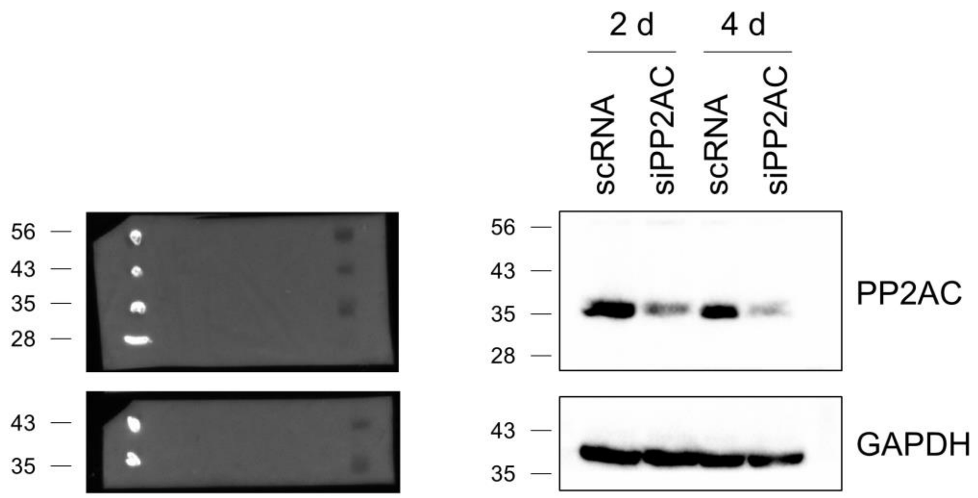

Supplementary Fig. 25 Uncropped immunoblots of **Supplementary Fig. 7c.**

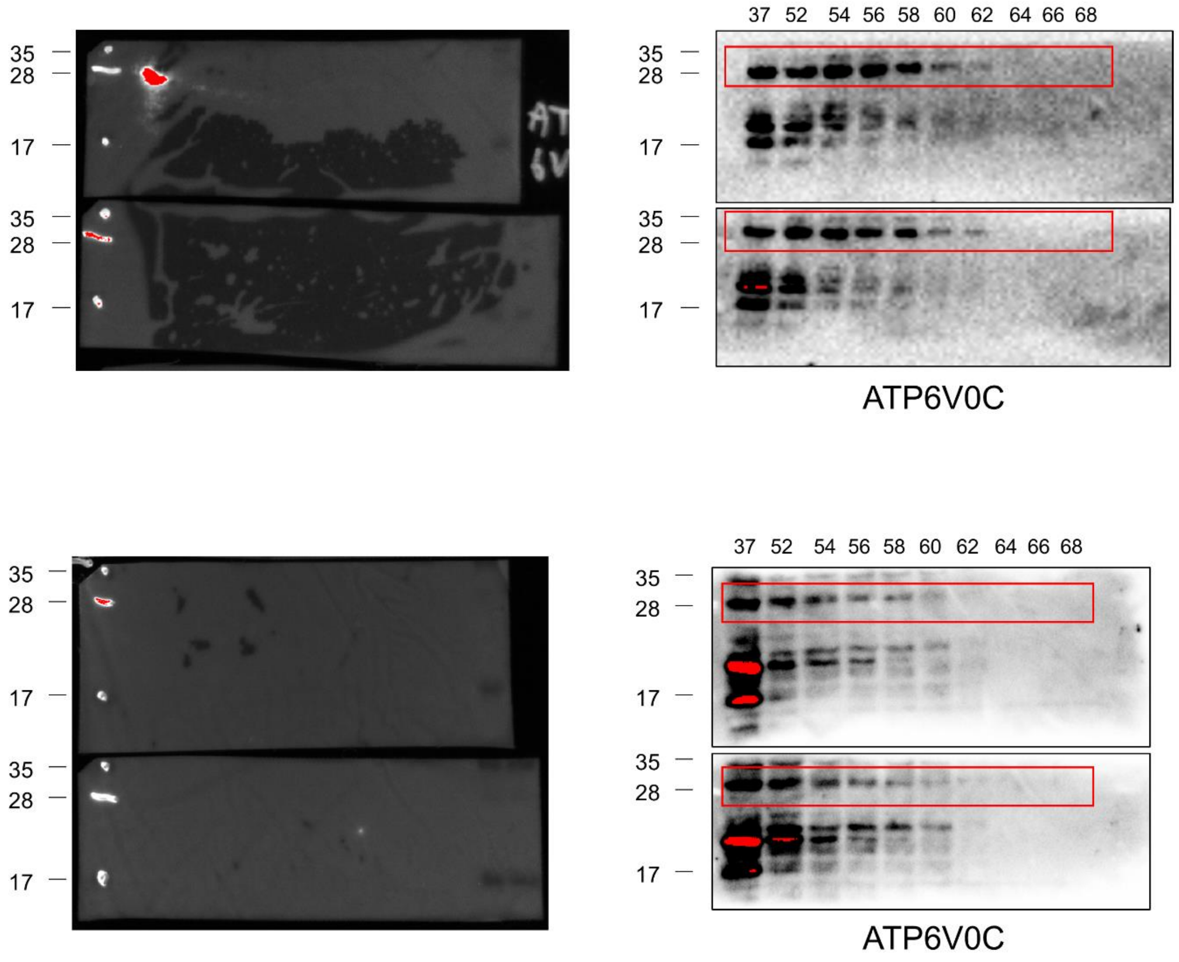

Supplementary Fig. 26 Uncropped immunoblots of **Supplementary Fig. 13.** Red rectangle means the cropped regions. Top: upper blot means DMSO-treated condition, and lower blot means CSG-treated condition. Bottom: upper blot means DMSO-treated condition, and lower blot means Baf-treated condition.

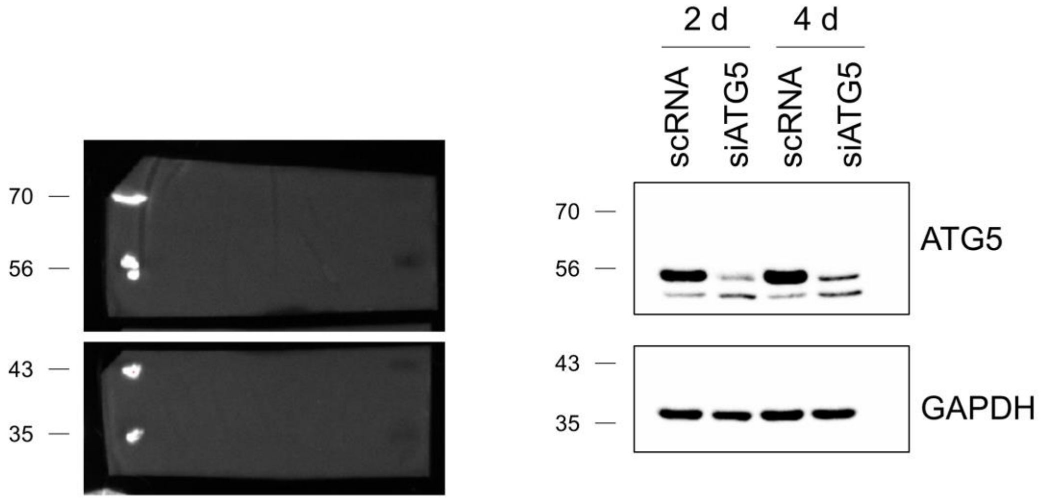

Supplementary Fig. 27 Uncropped immunoblots of **Supplementary Fig. 14c**

.

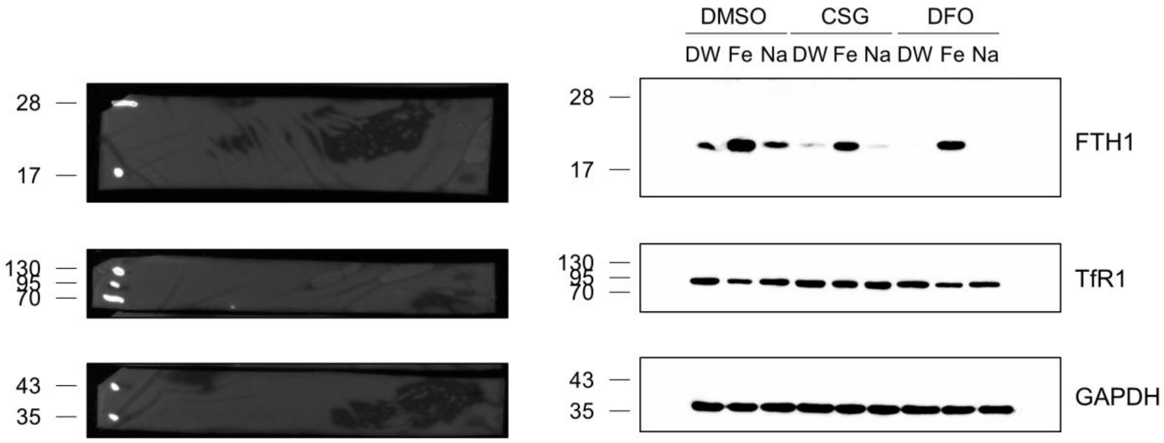

Supplementary Fig. 28 Uncropped immunoblots of **Supplementary Fig. 17a**

2. Supplementary Table

Protein spot		Matched to	Molecular weight	Mascot score	Quaries matched	Sequence coverage (%)	Name
Figure 2B	1G	DHSA_HUMAN	72645	261	20	26	Succinate dehydrogenase [ubiquinone] flavoprotein subunit, mitochondrial precursor (**SDHA**)
	1R						
	2G	AIFM1_HUMAN	66859	201	13	22	Apoptosis-inducing factor 1, mitochondrial precursor (**AIF**)
	2R	AIFM1_HUMAN	66859	316	24	32	Apoptosis-inducing factor 1, mitochondrial precursor (**AIF**)
	3G	ATPA_HUMAN	59714	1315	79	56	ATP synthase subunit alpha, mitochondrial precursor (**ATP5F1A**)
	3R						
	4R	UQCR2_HUMAN	48413	416	19	38	Ubiquinol-cytochrome-c reductase complex core protein 2, mitochondrial precursor (**UQCRC2**)
	4G	UQCR2_HUMAN	48413	591	32	49	Ubiquinol-cytochrome-c reductase complex core protein 2, mitochondrial precursor (**UQCRC2**)
	5R	UQCR2_HUMAN	48413	394	17	37	Ubiquinol-cytochrome-c reductase complex core protein 2, mitochondrial precursor (**UQCRC2**)
	5G						
	6G	TOM40_HUMAN	37869	188	11	22	Probable mitochondrial import receptor subunit TOM40 homolog (**TOMM40**)
	6R	TOM40_HUMAN	37869	199	13	29	Probable mitochondrial import receptor subunit TOM40 homolog (**TOMM40**)
Figure 2C	7G	PP2AA_HUMAN	35571	204	9	35	Serine/threonine-protein phosphatase 2A catalytic subunit alpha isoform (**PP2AC**)
	7R	PP2AA_HUMAN	35571	149	8	22	Serine/threonine-protein phosphatase 2A catalytic subunit alpha isoform (**PP2AC**)
Figure 2D	8G						
	8R	AT5F1_HUMAN	28890	136	9	31	ATP synthase B chain, mitochondrial precursor (**ATP5F1**)
	9R	NDUBA_HUMAN	20763	83	4	29	NADH dehydrogenase [ubiquinone] 1 beta subcomplex subunit 10 (**NDUFB10**)
	9G	NDUBA_HUMAN	20763	80	7	40	NADH dehydrogenase [ubiquinone] 1 beta subcomplex subunit 10 (**NDUFB10**)
Figure 2E	10G	BTF3_HUMAN	22154	114	15	66	Transcription factor BTF3 (**BTF3**)
	10R	BTF3_HUMAN	22154	232	16	77	Transcription factor BTF3 (**BTF3**)

Supplementary Table 1. Protein identification list obtained from the spot 1G/1R to 10G/10R in **Fig. 2b–e** by mass spectrometry analyses. See also **Supplementary Fig. 5** for further spot validation of SDHA (1G/1R), ATP5F1A (3G/3R), and UQCRC2 (4G/4R, 5G/5R).
