# Supplementary material for: Callyspongiolide kills cells by inducing mitochondrial dysfunction via cellular iron depletion
Source: Commun Biol. 2021 Sep 23;4:1123. doi: 10.1038/s42003-021-02643-8 (PMC8460830; doi:10.1038/s42003-021-02643-8)
Supplement: Supplementary file 3 — Description of Supplementary Files [file 42003_2021_2643_MOESM3_ESM.pdf]

**Description of Additional Supplementary Files**

**File name:** Supplementary Data 1

**Description:** All source data in main figures and supplementary figures.
