# Supplementary material for: Callyspongiolide kills cells by inducing mitochondrial dysfunction via cellular iron depletion
Source: Commun Biol. 2021 Sep 23;4:1123. doi: 10.1038/s42003-021-02643-8 (PMC8460830; doi:10.1038/s42003-021-02643-8)
Supplement: Supplementary file 5 — Reporting summary [file 42003_2021_2643_MOESM5_ESM.pdf]

nature research

Corresponding author(s): Prof. Seung Bum Park

Last updated by author(s): Aug 18, 2021

Reporting Summary

Nature Research wishes to improve the reproducibility of the work that we publish. This form provides structure for consistency and transparency in reporting. For further information on Nature Research policies, see Authors & Referees and the Editorial Policy Checklist .

Statistics

For all statistical analyses, confirm that the following items are present in the figure legend, table legend, main text, or Methods section.

n/a	Confirmed	
☐	☒	The exact sample size (n) for each experimental group/condition, given as a discrete number and unit of measurement
☐	☒	A statement on whether measurements were taken from distinct samples or whether the same sample was measured repeatedly
☐	☒	The statistical test(s) used AND whether they are one- or two-sided<br>*Only common tests should be described solely by name; describe more complex techniques in the Methods section.*
☒	☐	A description of all covariates tested
☐	☒	A description of any assumptions or corrections, such as tests of normality and adjustment for multiple comparisons
☐	☒	A full description of the statistical parameters including central tendency (e.g. means) or other basic estimates (e.g. regression coefficient) AND variation (e.g. standard deviation) or associated estimates of uncertainty (e.g. confidence intervals)
☐	☒	For null hypothesis testing, the test statistic (e.g. F, t, r) with confidence intervals, effect sizes, degrees of freedom and P value noted<br>*Give P values as exact values whenever suitable.*
☒	☐	For Bayesian analysis, information on the choice of priors and Markov chain Monte Carlo settings
☒	☐	For hierarchical and complex designs, identification of the appropriate level for tests and full reporting of outcomes
☒	☐	Estimates of effect sizes (e.g. Cohen's d, Pearson's r), indicating how they were calculated

Our web collection on statistics for biologists contains articles on many of the points above.

Software and code

Policy information about availability of computer code

Data collection	Cell viability was collected by measuring the absorbance with Gen5 [BioTek]. Fluorescent gel scanning was conducted with Sapphire Capture Software [Azure Biosystems]. Fluorescent cell imaging was collected with softWoRx [Cytiva]. FACS data was obtained with BD FACSDiva [BD Biosciences]. Immunoblot data were collected with Image Lab 4.0 [Bio-Rad]. qPCR data were collected with StepOne software v2.3 [Applied Biosystems]. No custom code was used in this paper.
Data analysis	Fluorescent cell images were analyzed with softWoRx [Cytiva] and ImageJ 1.53. Protein spot location and fluorescence intensity were analyzed with Melanie v9.2.3 [Cytiva]. All graphs were drawn with GraphPad Prism 8. Statistical comparison was conducted with GraphPad Prism 8. FACS data were analyzed with FlowJoX 10.0.7r2 software. No custom code was used in this paper.

For manuscripts utilizing custom algorithms or software that are central to the research but not yet described in published literature, software must be made available to editors/reviewers. We strongly encourage code deposition in a community repository (e.g. GitHub). See the Nature Research guidelines for submitting code & software for further information.

Data

Policy information about availability of data

All manuscripts must include a data availability statement. This statement should provide the following information, where applicable:

- Accession codes, unique identifiers, or web links for publicly available datasets
- A list of figures that have associated raw data
- A description of any restrictions on data availability

The raw numbers for charts and graphs are available in the Source Data file. All original data of this study are available from the corresponding author upon request.

Field-specific reporting

Please select the one below that is the best fit for your research. If you are not sure, read the appropriate sections before making your selection.

☒ Life sciences ☐ Behavioural & social sciences ☐ Ecological, evolutionary & environmental sciences

For a reference copy of the document with all sections, see nature.com/documents/nr-reporting-summary-flat.pdf

Life sciences study design

All studies must disclose on these points even when the disclosure is negative.

Sample size	No sample size calculation was performed. Sample size was chosen such that statistical significance could be confidently established.
Data exclusions	No data were excluded from the analyses.
Replication	All attempts at replication were successful.
Randomization	Not applicable, there was no experimental group allocation.
Blinding	Not applicable, there was no experimental group allocation.

Reporting for specific materials, systems and methods

We require information from authors about some types of materials, experimental systems and methods used in many studies. Here, indicate whether each material, system or method listed is relevant to your study. If you are not sure if a list item applies to your research, read the appropriate section before selecting a response.

Materials & experimental systems

n/a	Involved in the study	
☐	☒	Antibodies
☐	☒	Eukaryotic cell lines
☒	☐	Palaeontology
☒	☐	Animals and other organisms
☒	☐	Human research participants
☒	☐	Clinical data

Methods

n/a	Involved in the study	
☒	☐	ChIP-seq
☐	☒	Flow cytometry
☒	☐	MRI-based neuroimaging

Antibodies

Antibodies used

anti-caspase-3 (#9662; Cell Signaling Technology), anti-caspase-9 (#9502; Cell Signaling Technology), anti-PARP (#9532; Cell Signaling Technology), anti-SDHA (#11998; Cell Signaling Technology), anti-AIF (sc-13116; Santa Cruz Biotechnology, Dallas, TX, USA), anti-ATP5F1A (sc-136178; Santa Cruz Biotechnology), anti-UQCRC2 (sc-390378; Santa Cruz Biotechnology), anti-TOMM40 (PA5-57575; Invitrogen), anti-PP2AC (#2038; Cell Signaling Technology), anti-ATP5F1 (sc-514419; Santa Cruz Biotechnology), anti-NDUFB10 (PA5-51179; Invitrogen), anti-BTF3 (PA5-63299; Invitrogen), anti-ATP6V0C (PA5-23972; Invitrogen), anti-ATG5 (ab108327; Abcam, Cambridge, UK), anti-LC3B (#83506; Cell Signaling Technology), anti-p62 (ab91526; Abcam), anti-NDUFS1 (sc-271510; Santa Cruz Biotechnology), anti-SDHB (sc-271548; Santa Cruz Biotechnology), anti-UQCRFS1 (sc-271609; Santa Cruz Biotechnology), anti-FTH1 (#3998, Cell Signaling Technology), anti-TfR1 (#13113, Cell Signaling Technology), and anti-glyceraldehyde 3-phosphate dehydrogenase (#2118; Cell Signaling Technology), anti-rabbit IgG HRP-linked (#7074; Cell Signaling Technology, #7074), anti-mouse IgG HRP-linked (#7076, Cell Signaling Technology), anti-TOMM20 (ab186735; Abcam)

Validation

All antibodies were validated for their uses by the respective vendors.
anti-caspase-3: https://www.cellsignal.com/products/primary-antibodies/caspase-3-antibody/9662
anti-caspase-9: https://www.cellsignal.com/products/primary-antibodies/caspase-9-antibody-human-specific/9502
anti-PARP: https://www.cellsignal.com/products/primary-antibodies/parp-46d11-rabbit-mab/9532
anti-SDHA: https://www.cellsignal.com/products/primary-antibodies/sdha-d6j9m-xp-rabbit-mab/11998
anti-AIF: https://www.scbt.com/p/aif-antibody-e-1?requestFrom=search
anti-ATP5F1A: https://www.scbt.com/p/atp5a-antibody-51?requestFrom=search
anti-UQCRC2: https://www.scbt.com/p/uqcrc2-antibody-g-10?requestFrom=search
anti-TOMM40: https://www.thermofisher.com/antibody/product/TOMM40-Antibody-Polyclonal/PA5-57575
anti-PP2AC: https://www.cellsignal.com/products/primary-antibodies/pp2a-c-subunit-antibody/2038
anti-ATP5F1: https://www.scbt.com/p/atp5f1-antibody-c-12?requestFrom=search
anti-NDUFB10: https://www.thermofisher.com/antibody/product/NDUFB10-Antibody-Polyclonal/PA5-51179
anti-BTF3: https://www.thermofisher.com/antibody/product/BTF3-Antibody-Polyclonal/PA5-63299

anti-ATP6V0C: https://www.thermofisher.com/antibody/product/ATP6V0C-Antibody-Polyclonal/PA5-23972
anti-ATG5: https://www.abcam.com/apg5latg5-antibody-epr17552-ab108327.html
anti-LC3B: https://www.cellsignal.com/products/primary-antibodies/lc3b-e5q2k-mouse-mab/83506
anti-p62: https://www.abcam.com/sqstm1--p62-antibody-ab91526.html
anti-NDUFS1: https://www.scbt.com/p/ndufs1-antibody-e-8?requestFrom=search
anti-UQCRFS1: https://www.scbt.com/p/rieske-fes-antibody-a-5?requestFrom=search
anti-FTH1: https://www.cellsignal.com/products/primary-antibodies/fth1-antibody/3998
anti-TfR1: https://www.cellsignal.com/products/primary-antibodies/cd71-d7g9x-xp-rabbit-mab/13113
anti-GAPDH: https://www.cellsignal.com/products/primary-antibodies/gapdh-14c10-rabbit-mab/2118
anti-rabbit IgG: https://www.cellsignal.com/products/secondary-antibodies/anti-rabbit-igg-hrp-linked-antibody/7074
anti-mouse IgG: https://www.cellsignal.com/products/secondary-antibodies/anti-mouse-igg-hrp-linked-antibody/7076
anti-TOMM20: https://www.abcam.com/tomm20-antibody-epr15581-54-mitochondrial-marker-ab186735.html

Eukaryotic cell lines

Policy information about cell lines

Cell line source(s): A549, HCT116, HEK293T, HeLa, HepG2, Jurkat, MCF7, PC3, and SH-SY5Y were obtained from American Type Culture Collection.

Authentication: All cells were used without modification once received from the supplier, and therefore were not authenticated.

Mycoplasma contamination: All cell lines tested negative for Mycoplasma contamination.

Commonly misidentified lines (See ICLAC register): No commonly misidentified cell lines were used.

Flow Cytometry

Plots

Confirm that:

- [x] The axis labels state the marker and fluorochrome used (e.g. CD4-FITC).
- [x] The axis scales are clearly visible. Include numbers along axes only for bottom left plot of group (a 'group' is an analysis of identical markers).
- [x] All plots are contour plots with outliers or pseudocolor plots.
- [x] A numerical value for number of cells or percentage (with statistics) is provided.

Methodology

Sample preparation: Annexin V-PI staining: A day after A549 cells seeding on 12-well plate, the cells were treated with callyspongiolide (CSG, 200 nM) or etoposide (50 µM) for indicated times. The cells were trypsinized, and equal number of the cells in each condition was subjected to Annexin V-FITC apoptosis detection kit [Abcam, ab14085], according to manufacturer's instructions. Briefly, collected cells were suspended in the Binding buffer, then incubated with annexin V-FITC antibody and propodium iodide at room temperature for 5 min in dark. Fluorescently labeled cells were analyzed by FACS Aria II [BD Bioscience].
Cellular and mitochondrial ROS measurement: A day after A549 cells were seeded on 6-well plate, the cells were treated with ferric citrate (200 µM) or sodium citrate (200 µM) in the absence or presence of callyspongiolide (CSG, 200 nM) for 24 h. The media was aspirated, and the cells were washed with PBS. The cells were collected, suspended in PBS containing 2% FBS, and stained with 2′,7′-dichlorofluorescein diacetate (DCFDA, 10 µM) or MitoSOX (2.5 µM) for 30 min. The resulting cells were subjected to flow cytometry analysis using FACS Aria II [BD Biosciences].

Instrument: FACS AriaII [BD Biosciences]

Software: Flow cytometry data were collected using proprietary software packages BD FACSDiva [BD Biosciences]. The data were analyzed using FlowJo X 10.0.7r2 software [FlowJo, LLC, Ashland, OR, USA].

Cell population abundance: Flow cytometry to quantitate specific flourescence-labeled antibodies and flourophores but did not sort cells. Therefore post sort abundance is irrelevant.

Gating strategy: Annexin V-PI staining: annexin V-FITC was used to label apoptotic and necrotic cells, and PI was used to label necrotic cells. Vehicle-treated cells were stained with/without annexin V-FITC or PI respectively to determine gate.
Cellular and mitochondrial ROS measurement: vehicle-treated cells without DCFDA or mitoSOX staining were used to determine gate.

- [x] Tick this box to confirm that a figure exemplifying the gating strategy is provided in the Supplementary Information.
